# A Review on the Use of Hydroxyapatite-Carbonaceous Structure Composites in Bone Replacement Materials for Strengthening Purposes

**DOI:** 10.3390/ma11101813

**Published:** 2018-09-24

**Authors:** Humair A. Siddiqui, Kim L. Pickering, Michael R. Mucalo

**Affiliations:** 1 School of Engineering, Faculty of Science & Engineering, University of Waikato, Hamilton 3240, New Zealand; ahumair@hotmail.com (H.A.S.); klp@waikato.ac.nz (K.L.P.); 2 Department of Materials Engineering, Faculty of Chemical & Process Engineering, NED University of Engineering & Technology, Karachi 75270, Pakistan; 3 School of Science, Faculty of Science & Engineering, University of Waikato, Hamilton 3240, New Zealand

**Keywords:** hydroxyapatite, carbon, graphene, strengthening, toughening, fracture, crack bridging, nanotechnology, fracture mechanics

## Abstract

Biomedical materials constitute a vast scientific research field, which is devoted to producing medical devices which aid in enhancing human life. In this field, there is an enormous demand for long-lasting implants and bone substitutes that avoid rejection issues whilst providing favourable bioactivity, osteoconductivity and robust mechanical properties. Hydroxyapatite (HAp)-based biomaterials possess a close chemical resemblance to the mineral phase of bone, which give rise to their excellent biocompatibility, so allowing for them to serve the purpose of a bone-substituting and osteoconductive scaffold. The biodegradability of HAp is low (Ksp ≈ 6.62 × 10^−126^) as compared to other calcium phosphates materials, however they are known for their ability to develop bone-like apatite coatings on their surface for enhanced bone bonding. Despite its favourable bone regeneration properties, restrictions on the use of pure HAp ceramics in high load-bearing applications exist due to its inherently low mechanical properties (including low strength and fracture toughness, and poor wear resistance). Recent innovations in the field of bio-composites and nanoscience have reignited the investigation of utilising different carbonaceous materials for enhancing the mechanical properties of composites, including HAp-based bio-composites. Researchers have preferred carbonaceous materials with hydroxyapatite due to their inherent biocompatibility and good structural properties. It has been demonstrated that different structures of carbonaceous material can be used to improve the fracture toughness of HAp, as they can easily serve the purpose of being a second phase reinforcement, with the resulting composite still being a biocompatible material. Nanostructured carbonaceous structures, especially those in the form of fibres and sheets, were found to be very effective in increasing the fracture toughness values of HAp. Minor addition of CNTs (3 wt.%) has resulted in a more than 200% increase in fracture toughness of hydroxyapatite-nanorods/CNTs made using spark plasma sintering. This paper presents a current review of the research field of using different carbonaceous materials composited with hydroxyapatite with the intent being to produce high performance biomedically targeted materials.

## 1. Introduction

Significant numbers of people around the world have suffered from bone defects that are mainly due to trauma, bone-related diseases, and improper bone tissue growth. This situation has worsened during the recent era primarily because of a burgeoning global population suffering from a proportionately greater number of bone-related diseases, like age-related bone loss [1]. In addition, there are the effects of increasing sports-related injuries and traffic accidents, which have heightened the demand for bone tissue replacement [2]. At present, treatments for bone defects/substitutes include autografts and allografts, both having some important limitations. Limitations that are associated with autografts are mostly linked with a shortage of material available for autografting (i.e., from the iliac crest) and are also (rarely) linked with donor site morbidity. Disease transmission risks and poor immune response describe the main limitations associated with allografts. These limitations have provoked greater research efforts in bone tissue engineering with an aim to utilise a synergistic combination of different materials for functional bone regeneration [3]. In a typical bone tissue engineering protocol, a three-dimensional (3D) porous scaffold is initially made and is then loaded with specific living cells and/or tissue-inducing growth factors to initiate and promote tissue regeneration or replacement [2,4]. The initiative of bone tissue engineering has escalated research in the field of biomedical sciences, i.e., it has resulted in a more biologically focused and coherent way of designing and developing 3D scaffolds with an appropriate/desired porosity so that they can serve as reinforcement, support, and in some special cases, firmly establish tissue regeneration and replacement. A perfect scaffold is one that has an interconnected porous structure to guide new tissue in-growth and regeneration [2]. Large numbers of people, every year, find themselves in need of different kinds of biomaterials, like that needed for dental filling material and for hip joint replacements. After the procedure of blood transfusion, bone grafting has turned out to be the second most recurrently performed clinical procedure each year. However, the harsh reality is that many patients still accept the amputation of diseased/damaged bone/organ as an ultimate treatment, because of the unavailability of suitable bone graft substitute materials [5,6].

At present, various biomaterials are designed and fabricated using polymers, metals, ceramics, or their composites. Bioceramics and their composites have increasingly become an established class of materials applied as human body implants in the form of 3D scaffolds, as they have the necessary properties for biological activity in regard to cell adhesion, migration, and proliferation [2,4,7]. Amongst the different types of bioceramics available, those having a similar chemical identity to that of bone (i.e., calcium phosphate-based ceramics, like hydroxyapatite) have been found to be the most successful, however, their inherently low fracture toughness and strength have historically hampered their use in load-bearing applications [8,9,10,11,12].

This review intends to explore the ways in which the historically poor strength of pure hydroxyapatite implants has been improved through the use of different carbonaceous structures that have the potential to greatly enhance the strength of hydroxyapatite-based composites, manufactured either in the form of a bulk scaffold or as a coating. The article will describe the efforts that have been put together to present the abilities of different carbonaceous structures, especially those in nanoform, to favour crack bridging and deflection in relation to strengthening and toughening effects in a HAp matrix. The biomimetic roles of HAp and carbonaceous structures are also discussed in relation to developing a sound bio-mechanically active implant interface. These roles of carbonaceous materials provide for potential uses in many different biomedical related applications.

### Historical Perspective

In the past decades, scientists have focused their research efforts on designing and developing artificial bone, with the objective of creating functional bone regeneration candidates. The historical development of the various stages (“generations”) of biomaterials that resulted can be analysed through a consideration of their relative biomedical and mechanical properties. The first generation of biomaterials was mostly related with the development of high strength bioinert materials, which included the development of metallic materials (such as stainless steel and titanium alloys), ceramic materials (like alumina, zirconia), and polymeric materials (like polyethylene and silicone rubber). These biomaterials were used to make devices, which are commonly known as prostheses. At that time, scientists were only focused on an objective to achieve an appropriate combination of physical properties to match those of the substituted tissue (i.e., the defective site) with a minimal toxic response in the host [13]. Almost all of the biomaterials of the first generation had a common attribute, i.e., they were bioinert, and hence not able to develop a biological bond to bone or promote repair of bone. Moreover, they were non-degradable in vivo, which meant that secondary surgery was required if the implant failed and/or needed revision [6,7]. By the end of the 1980’s, approximately 50 types of implanted prostheses were in clinical use, which were made using 40 different biomaterials. Many people at that time had had successful surgeries, which saw their lives enhanced due to the use of implants made from ‘bioinert’ biomaterials. However, it was found (specifically in hip and knee implants) that over time there was a tendency for bone to become slowly resorbed at the prosthesis-bone interface, owing to a phenomenon that is known as “bulk modulus mismatch”. This is also known commonly as stress shielding, i.e., almost all of the mechanical load is carried by the higher modulus implant device, which initiates the localised osteoporosis-like symptoms leading to gradual bone resorption, deterioration of bone strength at the implant-bone interface, hence leading to ultimate failure via loosening. At that time, prominent materials scientists of the 1980s, such as Bonfield, began working on the concept of designing biomaterials that would diminish bone resorption due to stress shielding at the prosthesis-bone interface [14].

Later stages of research into biomaterials development were largely associated with advances in the field of artificial bone materials, which moved towards the use of bioactive or biodegradable materials. This era came to be known as that of the second-generation biomaterials. Bioactive ceramic materials (e.g., hydroxyapatite) and biodegradable polymeric materials (e.g., polylactic acid and polyglycolic acid) are important materials comprising the second-generation biomaterials group. Unlike the previous generation of materials, second-generation biomaterials possessed the property to develop strong bonds to surrounding bone tissue and they were biodegradable/bioresorbable. However, both generations of materials were clinically found to be merely functional/structural substitutes i.e., could only bear the load (in the case of the bioinert materials) or (in the case of the osteoconductive materials) provide a scaffold or “structure” in which bone formation had to be induced by natural bodily processes rather than by the implant itself. Some were also found to be insensitive to physiological changes in vivo, such as in the case of stress shielding by bioinert implants [5,11,14,15,16].

With the inception of multidisciplinary developments in the fields of molecular biology, materials science and manufacturing engineering, many innovative and advanced artificial substitutes having unique functionalities were developed, which are capable of bone repair/regeneration. This has led up to the stage where an important area of research known as tissue engineering was created [17,18,19,20]. Tissue Engineering develops scaffolds for placement in defect sites, which not only provide mechanical integrity but also encourage/actively induce stem cell proliferation. A typical procedure of tissue engineering consists of manufacturing a scaffold using suitable biomaterials and subsequently subjecting them to seeding/culturing of stem cells (already harvested from the patient) after which the scaffold is implanted. It can be applied in situations where a temporary functional repair is required or for permanent repair/regeneration. For a successful scaffold material, it is necessary to have an appropriate degradation rate, as very slow rates of degradation retard bone growth, while overly fast rates will result in loss of mechanical integrity. Therefore, it is necessary for a bone scaffold to possess the desired biological, mechanical, and topological properties, as well as favourable osteoconductive properties, thereby facilitating bone remodelling from artificial bone into natural bone [5,6,7,14,15,16,21].

In general, calcium, hydroxyapatite (HAp) based bioceramics show excellent biocompatibility, corrosion resistance, and very good compressive strength, which have made them a suitable candidate for implants. However, amongst the list of different calcium phosphates used biomedically, HAp has attracted the most interest from the scientific community due mainly to its features of promoting osseointegration and new bone formation processes, as well as its low toxicity, and resemblance to mineral bone. Research studies so far suggest that, among the list of calcium phosphate compounds, HAp-based materials have been the most successful in bone grafting applications.

## 2. Hydroxyapatite

The enhancement of advanced materials for biomedical applications is a critical issue challenging modern-day materials science and engineering, especially when it comes to the development of materials to be used in vivo. Historically, hydroxyapatite (HAp), Ca_10_(PO_4_)_6_(OH)_2_ has been the best bone substitute, because it can achieve a sound, firm bond with bone tissue, and furthermore, it can demonstrate osteoconductive behaviour and have no undesirable effects on the human body [15,22,23,24,25,26]. The term “hydroxyapatite” implies the presence of hydroxyl (OH) groups while “apatite” (derived from the Greek word “apatos”) meaning “to deceive” was historically used to identify mineral apatites, as they were often mistaken for precious gems, like topaz) [27]. Apatites, in general, are known by the chemical formula M_10_(XO_4_)_6_Z_2_, in which M^2+^ is a metal and XO_4_^3−^ and Z^-^ are anions. In the unit cell of HAp, the M^2+^ is Ca^2+^, XO_4_^3−^ is PO_4_^3-^ and Z^−^ is OH^-^. The Ca:P mole ratio in HAp is 1.67. HAp crystallises in a hexagonal system with crystallographic parameters: a = 9.418 Ǻ, c = 6.881 Ǻ, and β = 120°. The crystalline network of HAp is a compact assembly of tetrahedral PO_4_ groups, in which phosphorus atoms are found in the centre of tetrahedra having four oxygen atoms at the top. Each PO_4_ tetrahedron delineates two types of distinct channels. The first channel is surrounded by Ca^2+^ ions, denoted as Ca(I) (4 per unit cell). The second type of channel contains six other Ca^2+^ ions, denoted as Ca(II). These channels host OH^−^ groups along the c axis to balance out the positive charge [28].

**Figure 1:**
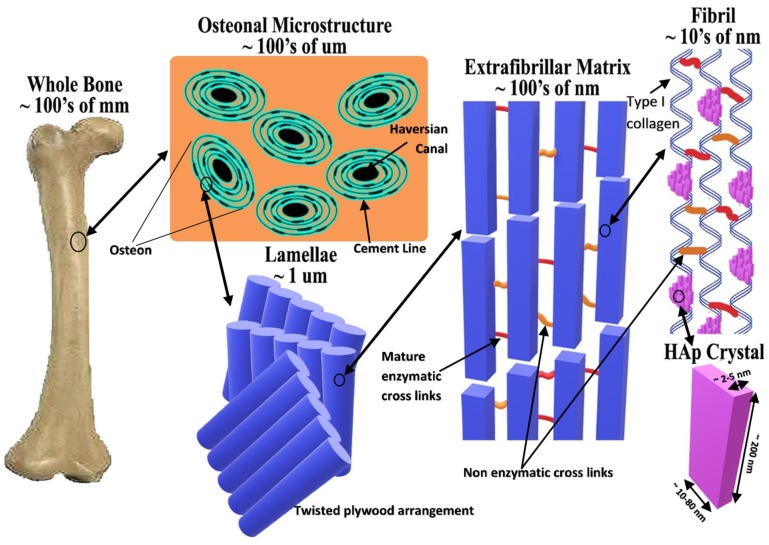
A microstructural representation of bone with size scales.

As long ago as 1926, diffraction and chemical studies of teeth and bone revealed that their inorganic phases were basically calcium hydroxyapatite [29]. In bone, the mineral phase consists broadly of hydroxyapatite but there is also a variety of impurity ions present in the HAp lattice, such as carbonate, magnesium, and sodium ions. Carbonate is one of the most abundant impurity ions with its content being about 4–8 wt.%. This is the reason that bone/hard tissue can be regarded as carbonate-substituted HAp (CHAp) [30,31]. Figure 1 represents the micron and sub-micron features of bone tissue, in which hydroxyapatite platelets can be seen. The biological behaviour of HAp-based ceramics relies on many factors, such as chemical and phase transformation, microstructure, pore size, and pore volume. In surgery, usage of both porous, as well as dense bioceramics, is common, as it depends upon the function and level of implantation that is required by the patient. It is most commonly dealt with on a case-to-case basis rather than in general. Experimentally porous ceramics have a low strength (although strength is found to be dependent upon the level of porosity), and hence such ceramics are found clinically to be more appropriate for drug delivery or for implantation into low load-bearing tissues (like maxillofacial applications) [22,32,33]. Osteointegration depends heavily on the pores in implants, more specifically, the pore size, volume, and their interconnectivity. It was observed that for bone ingrowth into an implant (successful osseointegration) the minimum pore size should be around 100–135 µm, and there is also found to be a direct relation between porosity/pore interconnectivity with the process of bony ingrowth into the implant, as the porosity/pore interconnectivity increases bone ingrowth, especially fixation processes, which become more operative. Moreover, protein adsorption favours separation of osteogenic cells. Subsequently, the presence of small, submicron pores, specifically those about the size of blood plasma proteins, favours biointegration. Thus, it is preferred to have a bimodal distribution of pore-sizes in bioceramics [20,23,33,34].

Small HAp granules are also of interest biomedically, as they find wide application in maxillofacial surgery and implantable drug delivery systems. There are numerous ways to produce HAp granules, like hydrothermal synthesis and pelletising. It was observed through scientific study that spherical HAp particles tend to promote osteointegration and diminish inflammatory processes [20,35]. Ceramics can also serve as a basis for producing composite materials. There is considerable research effort focused on synthesising HAp-matrix composites that achieve reinforcement of the ceramic materials through the use of fine particles, micro lamellae, or fibres, These can raise the strength and toughness of such materials to the level necessary for hard tissue (bone) replacement implants in more load bearing areas [24,36].

Researchers have synthesised hydroxyapatite using different techniques, such as hydrothermal synthesis, precipitation, and hydrolysis, as well as employing natural resources, like bovine bones [37,38,39,40], fish bones [41,42,43], marine shells [44,45,46], and eggshells [45,47,48,49]). Historically, the usage of xenogeneic bone, like Kiel bone (actual bone harvested from calf or ox) and Boplant (actual bone harvested from a calf) in biomedical applications, were thought to be the alternate to auto- or homografts, mainly due to limited supply of grafts, site morbidity, and the difficulty/expense of multiple surgical processes [22]. A study by Callan and Rohrer [39] also reported patient fears about human tissue transfer, due to the advent of AIDS, especially in dental surgery. They reported that clinicians were actively looking for alternative allograft materials and proposed a naturally derived xenogeneic hydroxyapatite (HAp). The xenogeneic bone grafting material that was used was a fully crystalline naturally porous bovine-derived hydroxyapatite (with all collagenous protein removed), having a particle size in the range of 250–450 µm. Although the organic matter had been previously removed, the bone microstructure was retained. Hydroxyapatite, either synthetic or naturally produced, has been used in wide variety of biomedical applications mainly due to its property of being able to form a bone-like apatite layer at the bone tissue interface [17,50,51]. As mentioned previously, despite these favourable bioactive characteristics of HAp, its poor mechanical properties, like low fracture strength and toughness, preclude its usage for high load bearing applications, such as an implant for the hip or knee. Therefore, to make HAp more suitable for such applications, it needs to be reinforced with various biocompatible materials for improving its strength and toughness. This hence necessitates HAp being prepared as a biocomposite material.

### Strengthening and Toughening of Hydroxyapatite—And Its Importance

**Figure 2:**
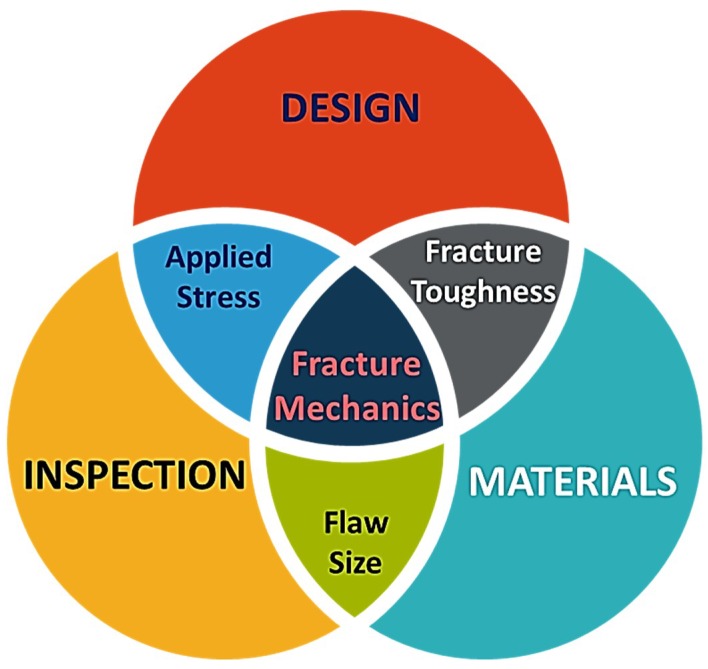
Picture depicting fracture mechanics as an integrated blend of applied stress, flaw size analysis, and fracture toughness.

The understanding and the application of strengthening and toughening mechanisms are very crucial for designing new and optimised materials. A common engineering goal for a structural material is to be both strong and tough, yet often, increase in strength is coupled with a reduction of toughness and vice versa. Strength is simply the material’s ability to withstand stress without being subjected to non-recoverable deformation or fracturing, whereas toughness can be regarded as the resistance to crack propagation [52,53]. Every material contains some form of defects (e.g., pores and cracks) and designing with brittle materials, such as alumina (defect-tolerant design philosophy), is challenging as they are influenced by such defects. Fracture mechanics is a valuable tool for design engineers, as it helps in identifying and evaluating conditions under which a crack/defect will propagate to cause a failure, usually by relating three important parameters i.e., the size of the largest/critical flaw, the applied stress, and the fracture toughness (Figure 2). Fracture toughness (or plane strain fracture toughness to use its full title) is a material property that can be obtained through experimental methods, like a three-point bending test, such that crack propagation is instigated. Design engineers can mathematically evaluate critical flaw size or critical stress levels using Equation (1) (fracture toughness equation), once the fracture toughness values are obtained.

(1) $$\;K_{IC}=Y\sigma \sqrt{\pi a}\;$$

where *K_IC_* is the fracture toughness, *Y* is the geometrical factor, *σ* is the applied stress, and a is the crack length [54].

Ceramic materials are known for their brittleness (lack of ability to plastically deform) [52,54,55,56]. In fracture mechanics theory (Griffith’s theory) fractures inside ceramic materials originate from micro-cracks rather than from atomic bond breaking [57]. Most importantly, these micro-flaws are omnipresent (microscopic flaws, including micro-cracks and internal pores, result from the cooling of the melt or during diffusion-based processes) in the body of a ceramic, and upon loading/stressing these microcracks tend to extend. Similarly, in the case of bioactive ceramics, despite the excellent biological properties, they lack slip systems in their crystals, so whenever they are subjected to an external load, stress relaxing phenomena, like plastic deformation and grain boundary sliding that can occur in metals, do not occur, resulting in poor load-bearing properties for such bioactive ceramics [6]. Hence, general failure mechanisms of ceramic materials are based on the unstable propagation of flaws (pores, cracks, or inclusions), as they are unable to relieve the stress build up at the tip of flaws (through plastic deformation, as, for example, a ductile metal would), and consequently, a ceramic body’s strength is dependent upon the combination of the size of the flaw and the applied stress. This explains why they are commonly referred to as notch-sensitive. The flaws in ceramics tend to act as stress concentrating points, i.e., they amplify the stress at the crack tip, which eventually leads to material failure at an applied stress point that is much lower than the theoretical stress [57] i.e., a significant decrease in the values of actual strength when being compared with theoretical values [57]. The theoretical strength of pristine glasses is about 7000 MPa, but practically their strength is merely 1% of the theoretical value (35–70 MPa) [58]. The dimensions (in micron or nanometre), geometry (penny-like round) and orientation (parallel to applied load) of cracks regulate the magnitude of this amplification of stress. This concentration of stress at the crack tip is commonly denoted by a stress concentration factor, which is the ratio between the maximum stress developed at the microstructural defect to the nominal stress in the body.

It is important to highlight the difference between the stress intensity factor and the stress concentration factor. The stress concentration factor, as defined earlier, is the ratio of maximum (which develops at the crack or flaw) to nominal stress in the body and it is dependent upon the geometry of the flaw or crack. Theoretically, the most important factor that is responsible for severe concentration of stress is a sharp crack (zero tip radius). In contrast, the stress intensity factor is used to predict the stress intensity near the crack/flaw tip that was developed due to remote or residual load or simply it evaluates/calculates the local driving force for crack propagation. This factor is dependent together on the geometry of crack and the applied load. The stress intensity factor is calculated for a given geometry and load and compared with a threshold value of stress intensity factor above which cracks will propagate in the given material. This threshold value of the stress intensity factor is known as fracture toughness or critical stress intensity factor. In Equation (1), the product “$\sigma \sqrt{\pi a}”\;$is the stress intensity factor, which represents how much the applied stress σ, gets intensified at the tip of the crack having a length a, so, technically, in brittle materials, for the fracture to occur, the stress that is developed at the crack tip must be greater than the fracture toughness values [54,55,59]. For bioceramics, where fracture events can be catastrophic, the approach based on utilising fracture toughness values, is the only method that can predict ceramic fracture.

When the stress developed at the crack tip exceeds the fracture toughness value of the material, the crack will fracture. It is important here to discuss the fracture process of this crack first, as the complete fracture is just a coalescence of the propagating crack with other ones. Fracture mechanics defines this process as a competition between two phenomena i.e., intrinsic (damage) processes and extrinsic crack-tip shielding mechanisms. Intrinsic damage processes, such as micro-void coalescence, promote the propagation of a crack tip by operating ahead of it and are dependent upon the nature of microstructure (or nanostructure) or any second phase ahead of crack tip (usually by cracking/debonding), while the extrinsic crack-tip-shielding mechanisms attempt to inhibit/resist this propagation of the crack tip and operate behind the crack tip. One of the principal methods of increasing fracture toughness is by increasing the microstructural resistance, which will enlarge the plastic zone ahead of the crack tip, eventually making initiation as well as propagation of the crack difficult. This is termed intrinsic toughening and is relevant to ductile materials. For ceramics/bioceramics, extrinsic toughening is perhaps the primary source of toughening, as intrinsic toughening would involve altering the bond strength, which is not feasible. Extrinsic toughening is generally based on diverse microstructural mechanisms that play their role (behind the crack tip) in reducing the damaging force developed at the crack tip; this is usually known as crack-tip shielding and can also involve processes like crack bridging by fibres and in situ phase transformation. Intrinsic toughening mechanisms affect crack initiation as well as crack propagation. They are an inherent property of the material and are found to be active in the material irrespective of the crack size and its geometry. Extrinsic mechanisms, which are only effective for crack growth, come in to play in the event of the crack wake and they are dependent on the size and geometry of the crack [52,53,60,61]. In the domain of biofabrication using bioceramics, designers are constantly challenged with the issue of low strength and toughness, for which extrinsic toughening is the only solution that is usually done by reinforcing bioceramics using biocompatible and effective materials [6,17].

Many successful approaches have been undertaken to toughen the ceramic material. The common strengthening and toughening mechanisms are:

1. Modulus/load transfer; i.e., the use of high elastic modulus fibres in a relatively low elastic modulus matrix [62].

2. Pre-stressing; which involves placing a portion of the ceramic under a residual compressive stress.

3. Crack shielding; mainly involving transformation toughening, in which reinforcement particles undergo sudden volumetric change due to an applied stress, which in turn, compresses the flaws in ceramic materials.

4. Crack deflection or impediment; mainly involving the modification in the microstructure of ceramic materials, like dispersing foreign particles, which tend to impede or deflect an advancing crack.

5. Crack bridging; implying the addition of some secondary phase, usually fibrous structures, which bridge the flaws in the ceramic, resulting in enhanced strength.

6. Fibre pull out; is associated with fibre debonding and the corresponding frictional sliding, which enhances the fracture toughness. Some part of the energy is consumed due to friction as fibre, particle, or grain slides against adjacent microstructural features, resulting in enhanced fracture toughness.

**Figure 3:**
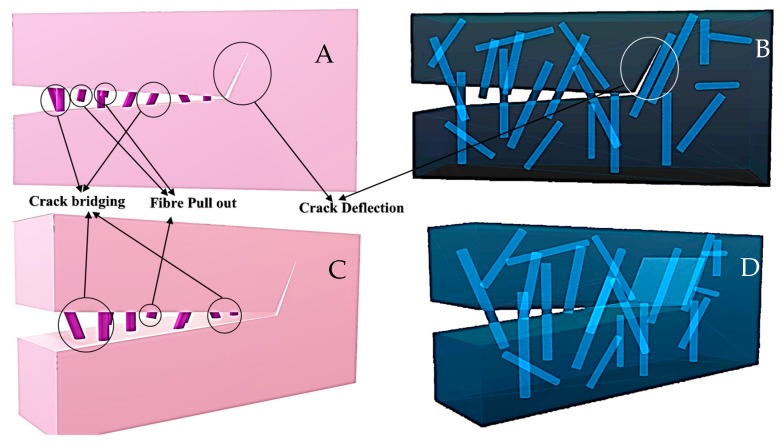
A typical representation of strengthening and toughening by fibres; (**A**,**C**) represent two views of crack in a body being perturbed by fibres, (**B**,**D**) represents the see-through image of the body presented in A and C, for a better understanding of the reinforcement effects.

**Figure 4:**
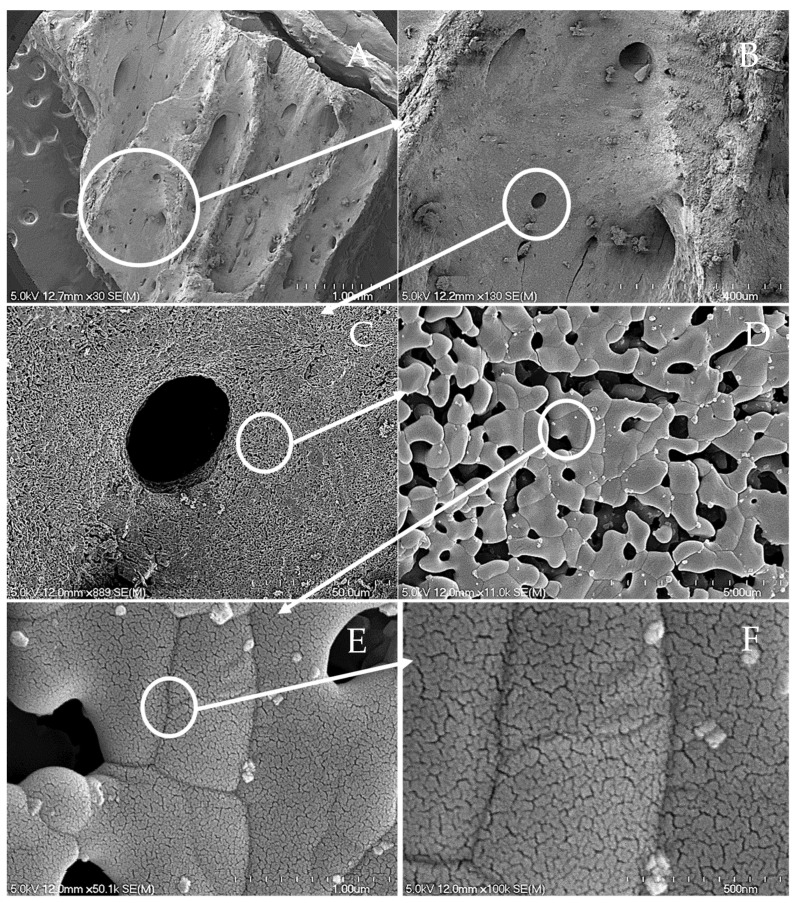
A series of SEM images of surface morphology of high temperature sintered bovine rib bone; (**A**) a particle of bovine bone-derived (CNF)/hydroxyapatite (HAp), (**B**,**C**) presents one of the interconnected pores, (**C**–**E**) presents surface imaging of a HAp particle & (**F**) depicts surface sub-micron cracks on the surface of HAp. These materials were generated by one of the review’s authors (Siddiqui).

Figure 3 represents an opaque and see-through image of the body containing fibrous reinforcement. These pictures clearly outline different strengthening and toughening mechanisms. Figure 4 represents a series of progressive SEM imaging of bovine (rib) bone sintered at high temperatures to burn out all of the collagen protein and lipid residues to yield crystalline hydroxyapatite.

## 3. Carbon and Its Structures

**Figure 5:**
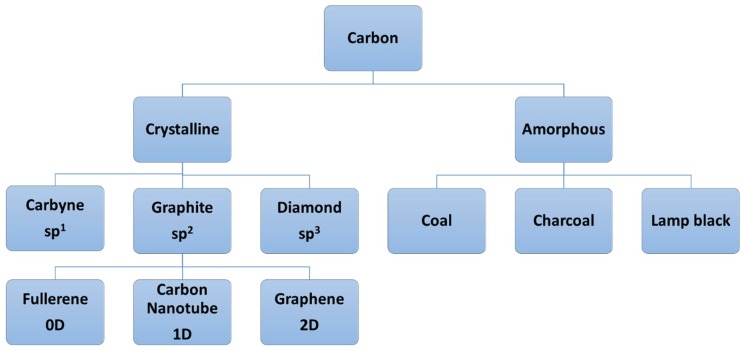
A broad classification of Carbon and its structures (0D, 1D and 2D means zero dimensional, one dimensional and two dimensional, respectively).

**Figure 6:**
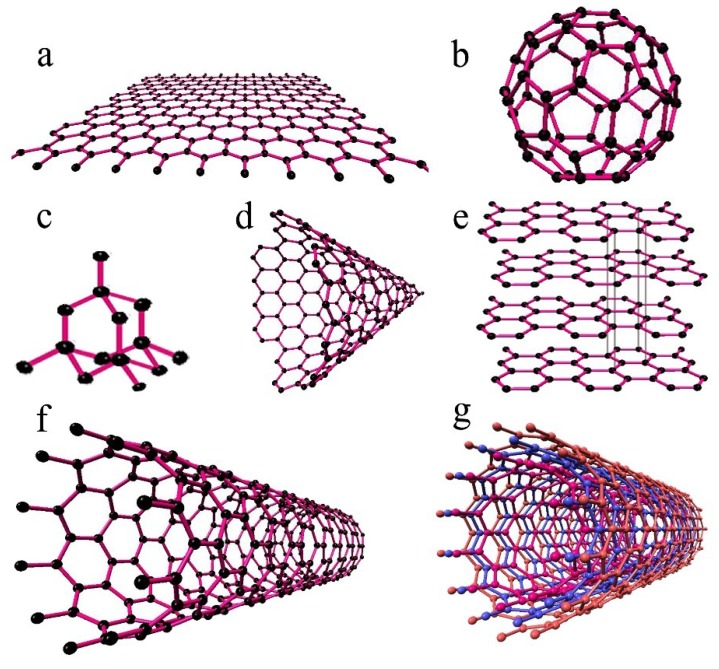
Some important carbonaceous structures, (**a**) Graphene, (**b**) Fullerene C_60_, (**c**) Diamond, (**d**) NanoCone, (**e**) Graphite, (**f**) Single-wall Carbon Nanotube, and (**g**) Multiwall Carbon Nanotube.

Carbon, one of the most abundant elements on earth, is the essential building block of all living organisms. The use of carbon/carbonaceous structures for biomedical applications is a novel trend as new diverse applications are continually being conceived. Carbon has many different allotropic forms and after hydrogen, is the material that can form the most compounds of any of the elements. Some well-known allotropes of carbon are graphite, diamond, glassy carbon, pyrolytic carbon, fullerenes (C_60_, C_70_), hexagonal diamond (lonsdaleite), and carbon nanotubes [63]. A broad classification of carbon allotropes is presented in Figure 5, while Figure 6 shows pictures of the different carbonaceous structures possible.

Carbonaceous materials have been finding ever-increasing applications in areas spanning the electronics, mechanics, civil engineering, and medical disciplines. Carbonaceous structures, especially carbon fibres, have a unique set of characteristics, namely, high elasticity modulus, as well as specific thermophysical, electrophysical, and sorption properties. This encourages the ever-expanding introduction of carbon composites into the most topical and knowledge-intensive branches of science and technology [64,65]. The biocompatibility of carbonaceous structures with native tissues of the body invites opportunities for medical applications (creation of artificial heart valves, the fixation of bone fractures, burns and diabetic ulcers, the creation of sorbing elements, and air filters). The creation of composites that are based on HAp and carbonaceous structures is a promising strategy for improving the mechanical characteristics of HAp-based implants [17,51,66,67,68].

Carbonaceous structures, if used in a limited/low quantity, tend to disperse more evenly and uniformly, leading them to develop good interfacial bonding within the ceramic matrix. It was observed in scientific studies that structures, like graphene and graphene oxide, were effective in enhancing fracture toughness, hardness, and wear resistance of ceramics, which is mainly due to their large surface area and wrinkled surfaces, as such surfaces would give rise to creative mechanical interlocking with the matrix [69]. Carbonaceous structures possess large elastic modulus (900–1000 GPa) values, which are significantly higher than those that were measured for bio-ceramic-constructed forms (40–50 GPa). As a result of this, when a crack propagates in these composite materials, the stress in the matrix can be efficiently transferred to the carbonaceous structures. At this point of crack propagation, carbonaceous structures will be subjected to stretching and fracturing at their ultimate strain values or will be dragged out from the matrix as the stress exceeds the critical interface bonding strength. In any of these scenarios, a significant amount of the fracture energy will hence be consumed. As the crack spreads further, a mechanism of crack bridging may start to appear, due to other carbonaceous structures being present in the matrix. The carbonaceous structures may start to act like ‘‘elastic bridges” to impede the further expansion of the crack [59,70]. In addition, to the bridging effect, there can be a crack deflection phenomenon operating, in which the crack interferes with the carbonaceous material, and instead of following its original path of propagation, it is deflected towards the ceramic/carbonaceous material interface. However, if the external energy is inadequate to debond the carbonaceous structure/matrix junction, the crack (crack tip) will become seized, i.e., a condition is achieved, like a shielding effect, which enhances fracture toughness by avoiding further fracturing of the matrix. These above-mentioned mechanisms make further propagation of a crack difficult as most of the external energy is consequently absorbed with little energy remaining for additional spread of the crack. Consequently, the crack spreads in a step-like pattern and progressively decelerates, or even ceases, thus increasing the fracture toughness. This provides a strengthened material capable of load-bearing applications. Therefore, introducing a secondary phase like a carbonaceous structure, which can form a strong interfacial bond with the ceramic materials, can play an effective role in impeding crack propagation and producing more robust and stronger bioceramics.

Optimisation experiments have indicated that the quantity of carbonaceous material is very important for mechanical properties, as when the content level approaches a certain threshold value, the optimum mechanical properties can be achieved, however, beyond that threshold value, mechanical properties begin to deteriorate [71]. This is attributed to the unwanted agglomeration of carbonaceous structures at high content levels, due to strong van der Waals forces, p-p stacking, and the deficiency of functional sites in carbonaceous structures. Apart from that, dispersion in a ceramic matrix is also inherently difficult, which can result in defects and weak interface bonding, eventually leading to a poor reinforcing effect. Hence, for achieving optimum properties of ceramic-based composites, optimisation of content, as well as the dispersion of the reinforcing phase, are all important requirements. So far, well dispersed carbonaceous structures/ceramic composite powders have been attained either by physical mixing or by colloidal processing. In a typical physical mixing, carbonaceous structures were initially ultrasonicated and then mixed with the ceramic powder by milling (usually ball milling) in a solvent. Ultrasonication of carbonaceous structure ensures disaggregation, which makes it suitable for mixing. In a typical colloidal processing protocol, both carbonaceous material and ceramic particles are modified in a way to induce opposite charges on their surfaces, due to which carbonaceous structures can then easily and effectively be dispersed in the ceramic powder via electrostatic attraction, albeit with ultrasonication and stirring being needed to effect efficient mixing. These modifications are brought about usually by surface functionalisation via acidification and oxidation or by using surfactants. As a result, through appropriate processing techniques, carbonaceous structures can mechanically strengthen a hydroxyapatite matrix via crack bridging and deflection mechanisms [6,57,72].

### 3.1. Pyrolytic Carbon

Pyrolytic carbon is highly fatigue resistant and it possesses elastic modulus values that are similar to that of bone. Historically, pyrolytic carbon was widely used for implant coating/construction, as such coatings tended to resist blood clotting on their surface and to make a frictionless surface, which is very useful in cases where the implant needs to have some sort of relative motion with other implants/surfaces. This material was also used for coating orthopaedic implants, like those that are used for finger joint implants, but was limited in their use in orthopaedic implants due to poor bone bonding properties [73,74]. Hetherington et al. [75] tried to investigate the bone response to HAp-coated pyrolytic carbon, as they plasma-sprayed HAp on pyrolytic carbon implants. Cylindrical samples of pyrolytic carbon, HAp-coated pyrolytic carbon, and HAp-coated titanium were implanted in the femurs of beagle dogs. After eight weeks, the beagles were euthanized, and the implants excised and investigated, which led to the discovery that the pyrolytic carbon had almost nil attachment strength (1.59 MPa), while the other two showed similar interfacial strengths (~8.71 MPa). This study showed that HAp favours a durable bond with the adjacent tissue. Similarly, Lin and Jiarui [73] have reported HAp coating on pyrolytic carbon via electrophoretic deposition (EPD) in glycol and ethanol separately (as the dispersion medium), as they were studying the effect of dispersing media on coating quality. However, this coating was characterised for its chemical and biological properties only, and not for its mechanical properties.

### 3.2. Carbon Fibres

Carbon fibres (CF) are crystalline filaments of carbon, having a regular hexagonal pattern of carbon sheets. Carbon fibres are widely used as a reinforcing material in polymer and ceramic matrices due to their high elasticity modulus, high strength-to-weight ratio, sorption, and thermophysical properties. The biomedical properties of CFs make them a very important material, as due to their inherent biocompatibility (both in vitro and in vivo), they have been used in making artificial heart valves, in treating bone fractures and purulent wounds, and in making biocomposites. They are usually manufactured via high-temperature conversions during the pyrolysis of carbon-rich (mostly polymeric) precursors [76].

N. A. Zakharova et al. [77] reported the successful manufacturing of composites comprising micrometre-sized carbon fibres (CFs) and biocompatible nanocrystalline calcium hydroxyapatite, containing 1.0, 2.0, and 5.0 wt.% CFs. This study was based on the principle of co-precipitation of HAp-carbon fibre with hydroxyapatite from a solution of calcium and orthophosphate ions and it was noted that the presence of carbon fibres noticeably affected hydroxyapatite crystallization from a system comprising Ca(OH)_2_–H_3_PO_4_–CF–H_2_O. Researchers found a progressive decrease in HAp sizes in response to increasing carbon fibre percentage, mainly because the presence of more carbon fibres meant more nucleation sites available, which indirectly lead to the formation of finer sized HAp particles. Slosarczyk et al. [78] manufactured a HAp—carbon fibre composite by hot pressing (using a temperature of 1100 °C, pressure of 25 MPa and an argon atmosphere). The composite was found to possess enhanced fracture strength and toughness. It was reported from this study that the effect of hot pressing and carbon fibre presence both played a substantial role in the strengthening and toughening effects that were observed.

Dorner-Reisel et al. [79] studied the microabrasion resistance of non-reinforced and CF-reinforced hydroxyapatite. The composite was made using commercial grade HAp and CF via hot pressing (using a pressure of 25 MPa, temperature of 1000 to 1150 °C, pressing time of 15 min, and an argon atmosphere). Apart from the chemical and structural characterisation, a ball crater test was utilised to ascertain the microabrasion resistance of the composite. It was observed that 20 vol.% of added CF increased the resistance of the composite against microabrasion. Cracking at the interface of the carbon fibres and the HAp matrix was observed after a wear test due to thermal mismatch. To prevent this, the investigators coated the CF with pyrolytic carbon.

Very recently, Boehm et al. [80] tried to investigate the full potential of reinforcing with carbon fibre by undertaking surface functionalisation of the CF. They worked on the concept of improving the wettability (which helps in precipitation) of a carbon fibre by activating their surface, which eventually leads to better adhesion of the fibre with the matrix. Fibres were modified using aqua regia and then calcium adhesion was performed while using CaCl_2_, which proved influential for the precipitation of calcium phosphate crystals on the fibre surface. This type of fibre modification process has great potential for making a strong composite for load-bearing applications.

### 3.3. Nano Carbonaceous Materials

Nano carbonaceous materials involve the use of several different types of materials with varied appearance and properties. Some important materials, for example, are carbon nanotubes, graphene, graphene oxide, and buckyballs. All these materials exhibit unusual electrical, mechanical, optical, thermal, and magnetic properties as compared to their bulk form, mainly due to quantum effects and large surface-to-volume ratios. Due to such unique properties, they are currently being used in a number of applications, such as in sensors and smart materials. When employed as second-phase materials for composites, nanotubes and sheet-like structures of carbon are successful due mainly to their large specific surface areas [69].

#### 3.3.1. Carbon Nanotubes

Carbon nanotubes (CNTs) are a type of one-dimensional nano-scaled material, having a tubular structure made by rolling single/multi-layer graphite sheets. CNTs, after their accidental discovery in 1991 by the Japanese physicist, Iijima, have piqued the interest of the scientific community mainly because of their unique structural and physicochemical properties. They possess large specific surface areas (50–1315 m^2^ g^−1^), a high aspect ratio (as they have their diameters at the nano-scale but their lengths, are, by contrast, at the micron-scale), high tensile strength, high resilience, and flexibility. The strength of CNTs is approximated to be roughly 100 times that of steel, but its density is just a fraction of that of steel. The reason for these highly favourable mechanical properties is due to the CAC (Carbon Atomic Chain) covalent bond that exists in the carbon rings, as these bonds are considered to be very stable chemical bonds. On the basis of this physical structure, carbon nanotubes can be single-walled carbon nanotube (SWNT) (single tube)—or multi-walled carbon nanotubes (MWNTs) (concentric cylinders of carbon) [71,81,82,83].

There are many different processes to prepare carbon nanotubes, however, arc-discharge [84,85], laser ablation [81,84,86,87], and chemical vapour deposition (CVD) [85,88,89] are the most common. For large quantities on a commercial scale, CVD is widely used for the preparation of CNTs. In a typical CVD process, the hydrocarbon feedstock is reacted with a metal catalyst at elevated temperatures to yield carbon nanotubes, however, the quality, quantity and dimensions of CNTs are heavily dependent on the reaction conditions. Owing to their unique properties, especially their property to act as carriers for bone morphogenetic proteins, they are regarded as highly successful candidates for reinforcing hydroxyapatite and numerous scientific studies have been performed to ascertain their strengthening and toughening effects in bio-ceramics. Most biomedical research using such composites shows that CNT-based biocomposites are appropriate for cell growth and enzyme activity [71,82].

Chen et al. [90] developed a strong CNTs-reinforced hydroxyapatite composite coating on titanium (Ti-6Al-4V) alloy. The composite coating was made while using a sophisticated technique known as “laser surface alloying”. Initially, commercial grade HAp and CNTs were mechanically ball milled in varying percentages of CNTs (0%, 5%, 10%, and 20%). A Nd: YAG laser, (output power 400 W) was used to apply the coating. After mechanical characterisation a significant increase in hardness was observed but only a slight improvement in modulus. In general, it was found that approx. 7 vol.% of carbon nanotubes increased fracture toughness by 50% and flexural strength by 28%. In comparison, Lahiri et al. [91] reported an increase of 92% and 25% in fracture toughness and elastic modulus values with the addition of 4 wt.% CNTs. Lahiri presented an increase of 260% in fracture toughness and of 50% in flexural strength in hydroxyapatite-nanorods/CNTs by the addition of 3 wt.% CNTs manufactured using spark plasma sintering. It was also reported that vacuum hot-pressing of hydroxyapatite ceramics also improved the mechanical properties [92].

Kealley et al. [93] developed a HAP-CNT composite via a chemical precipitation technique. Commercial CNTs (2 wt.%) were added during the precipitation stage of HAp manufacturing. The powder that was obtained was subjected to hot isostatic pressing at 900 °C under an argon atmosphere. In situ neutron diffraction was utilized to confirm that CNTs and hydroxide bonds were present in the composite. Kaya [94] developed a CNT-reinforced HAp coating, in which the addition of only 2 wt.% CNTs was found to significantly improve the hardness (i.e., by 10-fold), elastic modulus (by six-fold), and shear strength (by 3-fold) of the coating. Moreover, CNTs were also found to prevent spalling of the coating. CNTs were initially acid-treated to adjust the surface charge, which helped in the dispersion of CNTs in the HAp. Meng et al. [95] utilized nano-sized HAp particles with needle-like morphology to make a HAP-CNT composite via hot pressing. The composite was found to have enhanced mechanical properties. CNTs were initially functionalized to introduce -COOH groups on its surface, which helped in developing strong interactions between these and the Ca ions on the HAp particles. The composite demonstrated an approx. 50% increase in fracture toughness and a 28% increase in flexural strength with the addition of 7 vol.% CNTs. Sarkar et al. [96] utilized surfactant-modified CNT to make HAp-CNT composites via a spark plasma sintering process while using surfactant-modified CNT and HAp nanopowders. HAp was synthesised using a microwave-assisted process and was ball milled with 2.5 vol.% of CNTs. The resultant powder was spark plasma-sintered at various temperatures, where the temperature of 1100 °C produced the maximum fracture toughness of 1.27 MPa.m^1/2^.

Balani et al. [97] developed CNT-reinforced HAp coatings using plasma spraying. The powder feedstock for plasma spraying was comprised of 4% CNTs blended with HAp, which was sprayed on titanium Ti-6Al-4V substrate (power 20–25 kW). The coating was found to be highly crystalline and non-toxic with a ca. 56% improvement in fracture toughness. Li et al. [98] utilised a double in situ process to develop HAp-CNT composites. In the double in situ process, CNTs were initially synthesised and modified using HAp via CVD, and then were further encapsulated using HAp via a sol-gel process. The modification of CNTs with HAp proved to be helpful in developing a strong interfacial bond and a homogenous dispersion in the matrix, which led to enhanced biological as well as mechanical properties, as the flexural strength was found to be higher (1.6 times) than that of pure HAp. Kim et al. [99] reinforced HAp with different percentages of multiwalled carbon nanotubes and utilised the spark plasma sintering (SPS) technique for consolidation. Sintering at 900 °C, the composite attained maximum density, fracture toughness, and Vickers microhardness, but sintering beyond this temperature led to a degradation of the properties. It was also observed that increasing nanotube concentration was directly increasing the hardness and fracture toughness. A pull out mechanism was widely observed and declared to be the origin of the elevated fracture toughness values.

#### 3.3.2. Graphene

Graphene is a leading nanomaterial that commands a strong research following due to its diverse applications and properties. This material has also been of strong interest to biomaterial scientists due to its two-dimensional structure and large contact area, which makes it an ideal material for making composites. It is composed of a single layer of carbon (sp^2^-hybridized) atoms arranged in a honeycomb lattice-like arrangement. Graphene is the basic building block of all graphitic materials and possesses exceptional mechanical properties like tensile strength of ca. 130 GPa and a Young’s modulus ca. 0.5–1 TPa [100,101]. It has been found that graphene can stimulate a toughening effect in a HAp matrix even at a low content level; however, it would be appropriate to term this reinforcing graphene as “graphene nanosheets (GNS)”. This is because GNSs usually have thicknesses near 8–10 nm and are usually made up of a few graphene layers. GNS show similar properties to that of graphene (monolayers), in that they have strong mechanical, high electrical, and enhanced biological properties. From graphene can be derived two further important materials i.e., graphene oxide (GO) and reduced graphene oxide (RGO), which both have unique properties. Many biomaterial scientists have developed graphene-HAp based biocomposites for different biomedical applications, especially those that can be used for orthopaedic applications [102].

Liu et al. [103] developed graphene/hydroxyapatite composites using precipitation methods, as a result of which nucleation and growth of rod-like HAp nanoparticles on the graphene surface was observed, which occur most likely as the result of a charge balancing mechanism. After using arc spark plasma sintering, there was a significant increase in the fracture toughness (203%) with 1.0 wt.% graphene. In contrast, Zhang et al. [104] reported a maximum of 80% increase in the fracture toughness of HAp-1 wt.% CNT composite made via spark plasma sintering. This rise in fracture toughness was attributed to different strengthening and toughening mechanisms, like crack bridging, crack deflection, and pull-out effects. An in vivo study [105] on mice showed that the addition of graphene did not alter biocompatibility. Therefore, it is a very promising strategy to incorporate graphene as a second phase reinforcement in HAp, as it can enhance the mechanical properties of HAp without compromising the biological properties.

#### 3.3.3. Graphene Oxide

**Figure 7:**
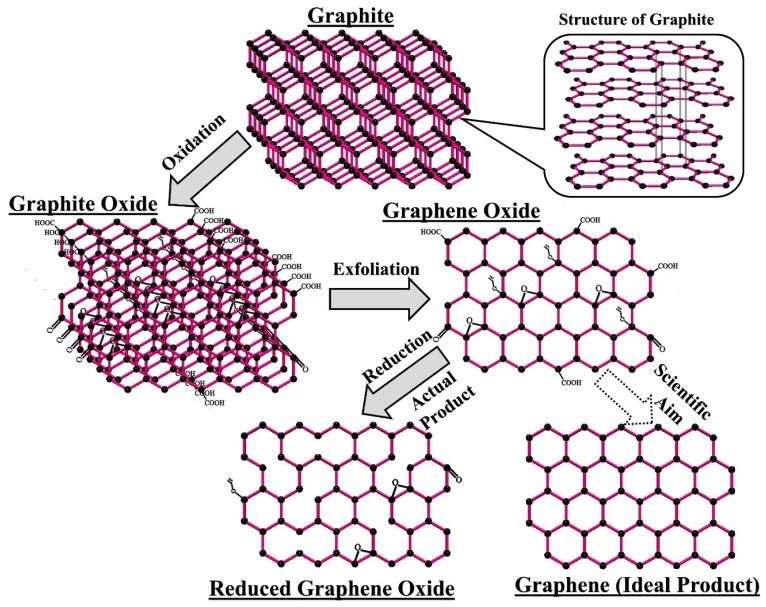
Scheme for making graphene oxide and reduced graphene oxide from graphite.

Graphene oxide (GO) is an important derivative of graphene. It can be referred to as the oxygenated counterpart of graphene as it consists of graphene sheets covered with oxygen-based functional groups, like hydroxyls (on its planes), epoxide (on its planes), and carboxyl and carboxyl groups (on its edges). Its particularly large surface area, biostability, biocompatibility, antibacterial properties, ease of chemical functionalisation, favourable dispersion behaviour, and mechanical properties are the main reasons for its widespread applications in diverse fields. The presence of functional groups on its surface facilitates in achieving dispersion stability and enhancement of interfacial bonding, which eventually leads to better load transfer in composites [106,107,108]. As compared to CNT, high-quality GO can be manufactured on a commercial scale at a lower cost. All of these properties and its relatively low cost of production have made GO an important material for nanoscale reinforcement in biocomposites. Several studies were conducted to synthesise HAp-GO based composites, as the incorporation of GO in HAp will ensure a good dispersibility and enhancement of mechanical strength. Historically, Schafhaeutl [109] had reported GO for the first time in 1840 (while working carbon for making cast iron, steel and malleable iron) and then Brodie [110] in 1859 (while discussing and evaluating the atomic density of graphite). At present, GO is mostly synthesised using a process proposed in 1958 by Hummers and Offeman [111] in which graphite is initially oxidised (using concentrated sulfuric acid, sodium nitrate, and potassium permanganate) to achieve GO having an increased interlayer distance addition of oxygen-containing functional groups. If this graphite oxide is subjected to ultrasonication, then it will dislodge the graphitic sheets to achieve graphene oxide (GO). There is also a modified Hummers method in which NaNO_3_ was eliminated to make the process safer and environmentally friendly, but the main strategy is the same. GO can also be reduced to achieve graphene, but due to partial reduction instead of graphene, “reduced graphene oxide” (RGO) usually results. Figure 7 depicts how GO and RGO can be obtained from graphite.

Li et al. [112] reported a significant reduction in surface cracks and an improvement in resistance to coating detachment in the HAp based coating by adding graphene oxide (GO) via a cathodic electrophoretic deposition process on a titanium substrate. Nano GO and HAp were commercially sourced and mixed together in varying percentages of GO (2% and 5%) via an ultrasonic process to ensure a homogenous distribution. The coating was achieved while using the electrophoretic deposition method with 30 V of constant voltage in a HAp-GO suspension. The coating was found to have superior corrosion resistance and in vitro biocompatibility, along with enhanced coating adhesion properties (ca. 75% increase with 2% GO and ca. 110% increase with 5% GO, when compared to the adhesion strength of pure HAp). Fathyunes and Khalil-Allafi [113] utilized ultrasound-assisted pulse electrodeposition to develop microstructurally refined and compact GO-HAp coatings on titanium substrates. The utilization of ultrasonic power (>60 W) was found to be effective for the incorporation/penetration of GO sheets into the coating. GO was synthesised using the modified Hummers’ method. The electrodeposition method was performed using an electrolyte containing Ca(NO_3_)_2_, NH_4_H_2_PO_4_, and H_2_O_2_, with the addition of a 100 μg/mL GO suspension, which resulted in the development of the GO-HAp coating. The complete process of electrodeposition was aided by ultrasonication with the HAp-GO coating being found to have higher hardness and modulus values (3.08 GPa of nano-hardness and 41.26 GPa of elastic modulus at the ultrasonic power of 60 W) together with enhanced corrosion resistance. Li et al. [114] successfully developed a GO-HAp composite using an in situ one-step mineralization method. GO was synthesised using the modified Hummers and Offema method. To perform the in situ mineralisation, GO and calcium chloride were ultrasonically mixed in a mixture of water and ethylene glycol and then disodium hydrogen phosphate solution was added, which stimulated the mineralisation of HAp, as confirmed by transmission electron microscopy (TEM), X-ray diffraction (XRD), Energy dispersive spectrometry (EDS), and Atomic Force Microscopy (AFM). Mechanical testing of GO-HAp and GO sheets/papers revealed higher modulus values (16.9 GPa), higher tensile strength (75.6 MPa), but reduced fracture toughness values (214.9 kJ m^-3^), as compared to GO alone. The modulus values of GO-HAp were found to be analogous to those measured for the human femur bone (13–15 GPa) and human tibia bone (13–16 GPa).

#### 3.3.4. Reduced Graphene Oxide

One attractive feature of GO is its ability to undergo (partial) reduction by removing oxygen-containing groups to yield graphene-like sheets. This recently developed material is a type of graphene and it has many terms describing it like “functionalized graphene”, “reduced graphene”, or “chemically modified/converted graphene”, but, in general, it is known as “Reduced Graphene Oxide” or RGO. The scientific aim of the reduction process of graphite/graphene oxide is to make pristine graphene, however, there always remain some defects and functional groups (residual) defects that can harshly alter the structure of the carbon plane. The reduction process can be chemical, thermal, or electrochemical. RGOs possess a honeycomb crystal lattice and they are found to have unique properties, like high electrical and thermal conductivity, biocompatibility, high surface area (>2600 m^2^ g^−1^), and chemical stability [108,115].

Liu et al. [103] developed HAp-RGO nanocomposites while using a liquid penetration technique. RGO was synthesised using the modified Hummer’s method and it was made into a suspension by dispersing in water. In the RGO suspension, HAp nanorods were wet-chemically synthesised to produce a HAp-RGO composite. HAp-RGO composite powders with varying percentages of RGO were pelletised using Spark Plasma Sintering. The composite’s fracture toughness values reached up to 3.94 MPa.m^1/2^; much higher than that for pure HAp. Crack tip shielding, crack deflection, and bridging were considered to be the reasons for the higher observed fracture toughness values, which were brought about by the inclusion of the RGO in the composite.

Baradaran et al. [116] developed HAp-RGO composites, in which HAp was used in the form of nanotubes. GO was prepared while using the simplified Hummer’s method, while HAp nanotubes were made using a surfactant-free solvothermal process. HAp-RGO composites were synthesised using a hydrothermal process during which GO was reduced to RGO then formed into the hybrid material. The powder was pelletised using hot isostatic pressing using 160 MPa pressure at 1150 °C. The composite showed an increase in the fracture toughness and elastic modulus of 40% and 86% respectively when compared to pure HAp. Biological testing revealed the promotion of osteoblast and proliferation activity on the composite. Similarly, Elif et al. [117] successfully reinforced HAp with RGO. RGO was made from GO, which was initially made using Hummer’s method, and then it was reduced using plant extracts to yield RGO. HAp-RGO composites were made using a liquid penetration method and were subjected to pelletising and sintering. It was observed that a small quantity (1%) of RGO had caused an increase of 3.2 times in the compressive strength of the composite as compared to the equivalent pure HAp sample. An increase in biocompatibility was also observed.

#### 3.3.5. Nanodiamonds

Nanosized diamond particles (Nanodiamond (ND)) are known for their mechanical, biological, and tribological properties, which include biocompatibility, high hardness, a low friction coefficient and chemical stability. This unique set of properties in ND has ignited intense interest for its applications as a secondary phase for reinforcing a bioceramic, especially a HAp matrix. On a commercial scale, NDs are formed by the detonation of carbon-based explosives and they are hence known as detonation nanodiamonds. In a detonation nanodiamond, there can be impurity species like O, N, Fe, Cr, Ca, and some functional groups like N–H, C–O–C, C–OH, C=O present which is given as the reason for the surface’s chemical multi-functionality. Nanodiamonds have also been used to produce mechanically strong HAp based coatings for load-bearing biomedical applications. The addition of nanodiamonds in HAp based coatings may also increase coating adhesion and prevent metal ion release from metal surfaces. Nanodiamonds have also been explored for their application in drug delivery applications [118,119,120].

Pramatarova et al. [121] developed and studied biomedical coatings that were based on hydroxyapatite reinforced with detonation nanodiamonds (DND). The coating was grown biomimetically using supersaturated simulated body fluids on different substrates (Ti, Ti alloy, glass). DNDs were created by the detonation of carbon-containing explosives and the generated shockwave at high temperatures and pressures, which produced very fine particles of DND (approx. size: 4-6 nm). These DNDs were added to an SBF solution, from which the growth of an HAp-DND coating was stimulated on different substrates. Upon comparison, it was found that the composite coating was more compact than that of pure HAp but was porous. Cell culture testing revealed DND was not toxic to living cells. Similarly, Chen et al. [122] synthesised a biomedical HAp–nanodiamond based coating and deposited it using plasma spraying on a titanium substrate. The nanosized HAp powder was synthesised using the wet chemical route, with the powder being subsequently added in 3.0 wt.% PVA to make the slurry. The ND suspension was made separately using commercial ND particles and a solvent (a 1:1 water/ethanol mixture). The coating was achieved via spark plasma sintering using a 0.5 wt.% and 2.0 wt.% ND suspension. The coating proved to be of uniform structure with low porosity and had enhanced mechanical properties when compared to the equivalent pure HAp-based coatings.

Similarly, Pecheva et al. [123] incorporated ND particles into SBF solution to make a coating on a titanium substrate using an electrodeposition method for which they used a three-electrode electrolytic cell. ND particles were made via a shock-wave propagation method and were added into an SBF solution. The coating obtained was found to be of a homogenous structure, free from residual stresses and possessing high hardness and ductility values. Li et al. [124] reported a novel approach in which NDs were first biofunctionalized by attaching bone morphogenetic protein 2 to them and then made a composite with HAp. Detonation NDs for this study were made using a shock wave propagation method and were then converted into a suspension (NDS-PBS (phosphate buffered saline)). The composite powder was coated using a vacuum cold spray method.

#### 3.3.6. Fullerenes

Fullerenes and their derivatives are found to have varied biomedical applications mostly in drug delivery systems. Fullerenes were discovered in 1985 and possess a carbon cage structure with immense scope for chemical derivatisation [125]. Fullerenes are entirely composed of carbon in the form of hollow spheres (or can be ellipsoidal in nature) or adopt related geometries. Highly symmetrical spherical fullerenes with icosahedral symmetry are known as Buckyballs or C_60_ [126]. Fullerenes are usually characterised by their hydrophobicity, three-dimensionality, electronic configuration, and good biological properties. The major issues related to the usage of fullerenes are its insolubility in aqueous media and aggregation [127]. One strategy to avoid such issues is chemical modification after which fullerene can be converted into fullerenol (a polyhydroxylated structure, C_60_(OH)_x_). This type of chemical modification is based on the addition of hydroxyl groups to the fullerene, which causes it to form into loosely associated, amorphous nano-aggregates. Djordjevic et al. [128] synthesised a hydroxyapatite/fullerenol nanocomposite via sonochemical processing; however, no mechanical characterisation was performed to access the strengthening effect. It was observed that the fullerenol nanoparticles had modified the surface of hydroxyapatite as the zeta potential values of the nanocomposite were ten times lower when compared to pure hydroxyapatite. This was attributed to the possibility of developing hydrogen bonding between surface phosphate groups in hydroxyapatite and hydroxyl groups in fullerenol.

#### 3.3.7. Carbon Nanofibres

Carbon nanofibres (CNFs) are linear sp^2^ carbon-based short fibres, having an aspect ratio greater than 100. They are generally vapour-grown or PAN (Polyacrylonitrile) based. CNFs possess excellent mechanical properties, biocompatibility, and non-toxicity, which render them suitable for reinforcement purposes in biomedical materials. Satoshi et al. [129] reported an increase of the mechanical properties in HAp based biocomposites due to carbon nanofibres (CNFs) reinforcement. The CNFs/HA composite was made via ball-milling techniques, followed by sintering using hot-pressing. It was observed that the addition of 10 vol.% CNFs resulted in a bending strength (i.e., 90 MPa), which was in the range of that of cortical bone. It was also found that the fracture toughness of CNFs/HAp composites was ca. 1.6 times higher than that of microporous HAp, but having the same level of bioactivity. Wu et al. [130] developed a carbon nanofibre (CNF)/hydroxyapatite (HAp) composite, having strong interfacial bonding and high mechanical strength. The CNF was made using carbonisation of electrospun polymeric (polyacrylonitrile) precursor nanofibres. CNF mat was alkali-treated to introduce carboxylic groups onto its surface, and was then immersed in SBF solution to precipitate HAp on its surface. The fracture strength of 41% CNF-reinforced composites was found to be 67.3 MPa.

## 4. Other Systems Involving Carbon Structures and Hydroxyapatite

Researchers in the past have explored the strategy of reinforcing hydroxyapatite with suitable carbonaceous structures; however, there are numerous studies in which some other components have been added into the composite for biological and/or mechanical enhancement of the composite.

Herkendell et al. [131] highlighted the issue of bacterial infections during bone surgeries and emphasized the addition of a bactericidal component in the biomaterial to deal with the issue. They developed a CNT-reinforced HAp composite containing small amounts of silver (Ag), which is known for its antibacterial properties. A HAp–4 wt.% CNT–10 wt.% Ag composite demonstrated high density and a maximum fracture toughness enhancement of up to 244%. The composite was found to have good anti-bacterial properties together with high interfacial strength. With the addition of different trace metallic elements, researchers have found significant improvements in the biological properties of the HAp-based bioceramics. These have exploited the fact that the HAp crystal lattice is labile to these cations [132,133,134]. Different bioactive metallic ions have been investigated with HAp based composites, like Sr^2+^, Mg^2+^, Mn^2+^, Ag^+^, Zn^2+^, and Y^3+^. Among these, for instance, Mn^2+^ was found to influence the osteoblast differentiation and bone resorption and to promote biological activity and the ability to promote bone. Similarly, Zn^2+^ was found to stimulate new bone formation, increasing bone density, inhibiting osteoclastic proliferation and bone resorption in vivo. Elements incorporated into HAp, such as Ag^+^ ions, need to be considered with some caution, however, because the presence of silver in vivo may lead to a medical condition known as Argyria in which permanent deposition of silver can occur in tissues causing cosmetic disfigurement if it is subcutaneous. This is discussed in detail in the paper by Hadrup and lam [135].

Chen et al. [136] developed a chitosan- CNT-HAp nanocomposite using an in-situ precipitation method. Chitosan is a fibre extracted from chitin, which develops in a crustacean’s exoskeleton. Chitosan is biocompatible and is biomedically exploited for various applications, such as bone tissue engineering [137,138,139,140] and drug delivery technology [141,142,143]. Multiwalled CNTs of 10 nm diameter were utilized and added into chitosan using ultrasonication. The composite was found to have good biocompatibility and also a maximum increase of ca. 110% in elastic modulus and 210% in compressive strength as the CNTs/chitosan wt. ratios rise from 0 to 5%. Yoon et al. [144] developed a gelatin-functionalised CNTs-HAp composite to mimic the natural structure of collagen fibrils in natural bone. In natural bone, collagen fibrils are interdigitated with HAp crystals and are responsible for strength and flexibility. Gelatine is a biocompatible, biodegradable, and high molecular weight polypeptide, which is already in medical usage, like wound dressings. Initially, in this reported research, a CNT/gelatin hybrid was made by covalently grafting gelatin molecules onto the surface of CNTs via the formation of amide linkages, then HAp crystals were assembled onto the CNT/gelatin hybridised surface. The structure of this composite is composed of CNT as a core and gelatin-HA as multi-layered shells. This hybrid material was found to be biocompatible and with enhanced mechanical properties.

Kalmodia et al. [145] manufactured a HAp -Alumina (Al_2_O_3_) -multiwalled carbon nanotubes (CNTs) composite. Previous, separate studies showed an enhancement of fracture toughness of the HAp matrix using individual Al_2_O_3_ reinforcement [146,147] and CNT reinforcement [72,93,98,99]. In this study, researchers were interested in investigating the combined reinforcing effect of CNT and Al_2_O_3_ in a HAp matrix. The composite was made while using spark plasma sintering and it was found to be biocompatible and also demonstrated favourable cell adhesion and proliferation properties. The highest hardness was recorded in a HAp-Al_2_O_3_ sample, but enhanced fracture toughness was observed when CNTs were added. Khanal et al. [72] utilised nylon along with carboxyl-functionalized CNTS (single walled) in a HAp matrix to enhance fracture toughness. The process comprised precipitation of HAp particles, which were then mixed with predefined quantities of CNTs and nylon. The fracture toughness values of the 1 wt.% carboxyl-functionalised single-walled CNTs and nylon were found to be 3.60 MPa.m^1/2^.

Murugan et al. [148] found an enhancement in the biological and mechanical properties of mineralised hydroxyapatite reinforced by GO and oxidised CNF. GO was made using Hummer’s method, while commercial CNF was treated while using NaOH. Among the different percentages trialled, 1% oxidised CNF and 1% GO-based composite were found to have the best properties for orthopaedic applications, like mechanical strength, which was 468 ± 4 HV (by Vicker’s micro-hardness), along with favourable bactericidal properties.

## 5. Biomimetic Role of Hydroxyapatite and Carbon Composite Materials Intended as Biomaterials

**Figure 8:**
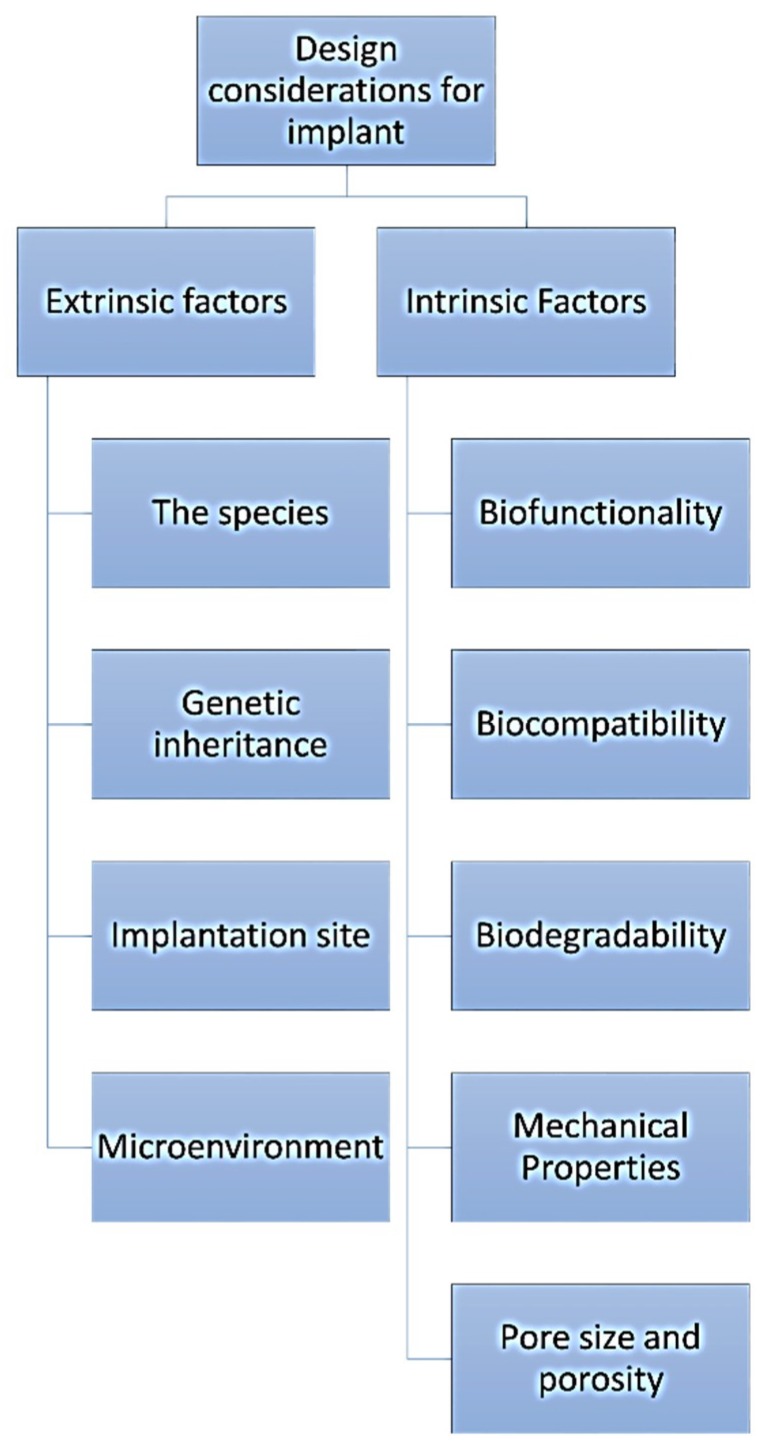
A general classification of several factors needs to be considered when designing an implant material.

Gustave Eiffel designed The Eiffel Tower after being inspired by the load distribution features from observing the “trabeculae” of human thigh bone (the longest and strongest bone in the body). In the biomedical field, using knowledge that is gained from biomimetics and biomineralisation can lead to biomaterials that are closer in function to natural boney tissues [149]. For an ideal implant material that can provide a natural environment for surrounding tissues and cells, it is necessary to meet the biochemical requirements of bone tissue engineering and to have the necessary mechanical properties to provide a framework for surrounding cells/tissue to interact with its surface. The environment leading to new tissue growth should eventually lead toward bone remodelling and new bone formation [16,150,151,152]. Designers for implant material(s) need to consider several different factors, which can either be extrinsic or intrinsic (Figure 8), however most of the extrinsic factors are outlined by medical professionals. An implant material is anticipated to complete the process of bone regeneration and healing after which implant removal becomes desirable from a clinical and biomechanical point of view. So, in such situations, bioactive and biodegradable materials are required to avoid a second surgery to remove them. Bioactive and biodegradable ceramics possess the unique ability of developing a bond to bone tissue, making them useful as 3D scaffolds and coatings [16,151,152]. Some of the most researched bioactive ceramics for bone augmentation are hydroxyapatite [34,153], β-tricalcium phosphate (β-TCP) [154,155], and bioactive glass [156,157,158]. They possess different rates and extents of resorption, however, all of them are biocompatible and osteoconductive.

Hydroxyapatite is found to have low biodegradability due to its low solubility (Ksp ≈ 6.62 × 10^−126^) and it develops a bone-like calcium phosphate coating on the implant surface, which helps in bonding to the surrounding tissues [159,160]. Kim et al. [161] demonstrated how electrostatic interactions of the HAp surface, with Ca^2+^ and PO_4_^3−^ ions promotes bone like apatite formation. The study was carried out using SBF. When an HAp surface is exposed to SBF, the surface displays negative charge and interacts with the Ca^2+^ ions, thereby forming a Ca-rich amorphous calcium phosphate layer, which resulted in the alteration of charge i.e., now the surface become positively charged. The process proceeds with the electrostatic interaction with PO_4_^3−^ ions and forms the Ca-deficient amorphous calcium phosphate, which ultimately matures into bone-like apatite. Brandt et al. [162] reported hydroxyapatite implantation in rabbit femoral bone for 12 weeks, in which it was found that HAp exhibited bone formation, minimal degradation, and slow resorption. Following implantation, serum proteins are known to be readily adsorbed on the implant surface, which favourably alters the interfacial properties of the scaffold leading to initial in vivo resorption mostly from cellular activity. Tissue remodelling (bone formation and resorption) is carried out by osteoblast (bone forming) and osteoclast (bone resorbing) cells. When a hydroxyapatite-based implant is inserted into the physiological environment, a foreign body response is initiated, which follows a series of different phenomena, namely: injury (surgeons initially have to “injure” certain tissues and remove them to some extent to fix implant), protein matter interaction, blood clotting, inflammatory responses, fibrous tissue formation, and tissue remodelling. The resorption of HAp in the physiological environment creates space for newly developing tissues, which not only grows along the surface of the implant, but also infiltrates into the scaffold. This infiltration is accompanied with blood vessels (and oxygen supply for regenerating tissues), which eventually allows bone formation to progress. The initial degradation of HAp-based implants is highly dependent upon its properties, like porosity, surface roughness, and the site of implantation, as they directly affect the phenomena occurring near the implant surface (fluid exchange and nature of surrounding medium) [152]. Moreover, one of the most important parameters for bone augmentation is the volume of new bone formed, which is highly dependent on an adequate blood supply. Foreign body response near the implant results in a different physiological environment as there is found to be enhanced concentrations of reactive oxygen, proteolytic enzymes, fibrotic proteins, giant cells, as well as a reduced pH in the vicinity of the implant. The osteoinductive properties of bone-like materials are largely dependent upon calcium (Ca^2+^) and phosphate (PO_4_^3-^) ions, as during in vivo bone resorption, osteoclasts resorb calcium (Ca^2+^) and phosphate (PO_4_^3-^) ions from bone matrix, which results in a local increase of ion concentration. This ionic gradient assists in bone formation by the proliferation and differentiation of osteoblast cells. Then, the presence of interconnected macro and micro porosity (and its distribution) in HAp-based implants plays a significant role in boney tissue ingrowth, as it is responsible for oxygen and blood exchange during cell and bone growth [160].

For designing an effective biologically active surface in an implant material that can orchestrate these physiological healing process, the strong effect of altered environment near the implant is considered. In general, cellular response, which includes cell adhesion, proliferation, migration, and differentiation, is heavily influenced by the implant’s surface chemistry, surface finish (the degree of roughness), topography, and wettability. There are several mechanisms responsible for effective cell adhesion to a biomaterial surface, including weak van der Waal’s forces and electrostatic attraction; mechanical clinching to surface topographical structures and specific interactions between cell surface receptors and specific ligand molecules, as different cells react differently to the various surface features [16,151,163]. Jarcho [164] reported after clinical studies that HAp could develop direct physiological bonding to bone, with good biocompatibility and no inflammatory response. Initially, the surface undergoes partial solubility, which leads to direct deposition of calcified bone matrix. In vitro studies have shown that the presence of micro and macro porosities in HAp influence the degradation behaviour of HAp, but not the bonding of bone tissue. Wenisch et al. [165] studied the degradation of hydroxyapatite implanted in a sheep for six weeks, via transmission electron microscopy. The degradation was found to be osteoclast-mediated. Guda et al. [166] conducted a clinical animal study to find the effect of pore size on bone regeneration and reported that large uniform pore sizes result in enhanced bone regeneration.

Similarly, in the case of carbonaceous matter included in biomedical materials, their properties are mostly related to their surface features. Rajzer et al. [167] reported microscopic studies both in vivo and in vitro on two types of carbon fibres, in which one was HAp-modified, while the other type was a porous carbonaceous material. It was found out that cell adhesion was more effective on porous fibres when compared to the HAp-modified carbon fibres, which is most likely due to the increased surface area. Similarly, the significant amount of porosity also enhanced the tissue and cell growth within the implant structure, which has been observed to be the case with porous mineralized structures. As far as the biocompatibility of carbonaceous structures, especially carbon fibres, is concerned, it is known to be well tolerated by the body without any foreign reaction. More interestingly, the newly developed bony structure on the carbon scaffolds was found to be morphologically and functionally similar in form to the replaced natural bone. However, it was observed that in the case of fibrous carbon the cellular responses were inversely dependent upon the crystallinity i.e., amorphous fibres were judged to be excellent for implants rather that high crystallinity ones. Some studies [168,169] have revealed the adequate haemocompatibility; reduced thrombus formation in amorphous carbon. Du et al. [170] reported osteoblast cells attachment and proliferation on the surface of diamond like carbon (amorphous carbon) that was deposited on silicon substrates. Perkin and Naderi [171] reviewed and declared carbon nanostructures safe for clinical uses. They observed that, if the carbon structure is fixed in a solid composite structure, the effect of toxicity is reduced. Moreover, if the nanostructure detaches from the solid composite, then functionalisation will prevent its bio-accumulation. Schipper et al. [172] reported a clinical study in which functionalized-SWCNTs were injected into the bloodstream of mice. No toxic effect was noted for four months following treatment. Usui et al. [173] reported bone regeneration properties of MWCNTs, along with high bone tissue compatibility, as stimulated by human bone protein. Blazewicz [174] reported that highly carbonized (more crystalline) carbon fibres showed gradual fragmentation in biological environment as compared to low carbonised carbon fibres (i.e., with smaller crystallite sizes), which were easily resorbed and integrated in the body. Mild toxicity was also reported for CNTs mainly due to oxidative stress and inflammation [175]. Peterson [176] conducted an animal study on epoxy/carbon fibre-composite and reported that carbon fibres can undergo osseointegration with live bone.

There is a continued interest in making biocomposites with exceptional bone bonding abilities, for which both the scaffold matrix and its reinforcement are selected very prudently. The essential property that is required from a biocomposite is to demonstrate the formation of bone-like apatite on its surface. For designers, biocompatibility is not the only important parameter to consider for an ideally functioning implant material. It is also the strength, distribution of reinforcement, surface chemistry, porosity, microstructure, and surface roughness that comprise the other considerations related to the implant material. When a biocomposite is made using hydroxyapatite and carbonaceous structures (mainly for the purpose of overcoming the strength issues of hydroxyapatite), it is required that the resulting composite should also maintain adequate bone bonding properties, as otherwise the purpose of using a biologically inspired implant is incomplete. Hence the role of the reinforcing carbon is to maintain or minimally disrupt these bioactive properties of hydroxyapatite. As reported earlier, researchers have added different carbonaceous structures into hydroxyapatite, like nanocarbon forms, diamond and each resulting composite were found to possess different properties. In general, it is agreed that the carbon is bio-inert and has no toxic effect on living tissues [177]. Researchers have also evaluated the combined effects of HAp and carbon, either in the form of solid composites or as coatings. Mostly, nanostructures of carbon with HAp were evaluated for their biological properties. Khalid et al. [178] studied the interaction of functionalized-MWCNTs (f-MWCNTs) and f-MWCNTs-reinforced HAp composite with human osteoblast (sarcoma cell lines) in vitro. No damaging effect was noted on the survival of osteoblast cells, whereas it was noted that an increasing concentration of functionalized-MWCNTs had a cytotoxic effect. In a similar in vitro study, Khalid and Suman [179] reported no detrimental effect of f-MWCNTs HAp nanocomposites on a mouse fibroblast cell line. Liu et al. [180] demonstrated enhanced biocompatibility and cell growth properties by modifying pure carbon nanofibres using calcium phosphate. Similarly, Huang et al. [163] developed homogenous nanostructured calcium phosphate coatings on chemically modified carbon fibres using a biomineralisation process, which had promising biological properties. Han et al. [181] enhanced cell attachment and proliferation by modifying carbon fabric with calcium phosphate. Chlopek et al. [182] reported a modification of a carbon-carbon composite using hydroxyapatite powder. Upon testing them in an artificial biological environment, it was observed that the composite with a HAp-enriched surface provided favourable conditions for bone apatite growth. Newman et al. [183] developed a high porosity mechanically strong composite while using β-tricalcium phosphate/hydroxyapatite. A high-quality coating that is based on CNTs was applied to the composite for biomedical applications. Martinelli et al. [184] reported promising bone regeneration properties of nano HAP-CNT thin films developed on biomedical stainless steel via an in vitro osteogenesis study. Murugan, Murugan and Sundramoorthy [185] developed a biological coating based on hydroxyapatite/polycaprolactone-graphene oxide on Ti alloy. In vitro testing of this coating revealed outstanding cell viability, while in vivo testing in a rat model revealed bone formation after 28 days of implantation.

## 6. Conclusions and Future Aspects

In this review, the reinforcing effects of different carbonaceous structures in a HAp matrix (as scaffolds or coatings for orthopaedic applications) were discussed. The current demand on bone tissue engineering is to produce a scaffold or coating that should by itself be strong enough to bear loads, and to also possess favourable biocompatibility and bioactivity properties. This objective can be analysed in two parts as the first one deals with the mechanical properties, while the other deals with the biomedical/biocompatibility properties and out of the two, the maintenance of good biomedical properties is of the utmost importance i.e., it is crucial to select a material having exceptional biomedical properties, and then it is necessary to evaluate its overall strength and toughness. To produce such a robust material, scientists mostly rely on apatites/hydroxyapatite because of the highly favourable biomedical properties of these ceramics and then need to take steps to enhance its strength and toughness. However, reinforcement to enhance mechanical properties must be done without any compromise to the biological properties. Out of the many different materials, which can enhance the mechanical properties of HAp matrix, carbonaceous structures are always preferred in the biomedical science community because of their strength and biocompatibility. In this paper, it was highlighted that different structures, quantities, and modifications of carbon can be useful in increasing the strength and toughness of the HAp matrix. The biodegradability, osteoconductivity, and interface features of implant materials were also discussed, which suggests that the strategy to reinforce carbonaceous structure within the matrix of HAp will not disturb the bone regeneration properties of the composite.

As for future prospects, it is apparent that previous researchers have been focussed on achieving the maximum strength in a HAp matrix with use of the minimum amount of reinforcing the material, for which nano-reinforcements or nanofillers are a suitable choice. Moreover, it can also be observed that the morphology of reinforcing materials is also very important as in the case of nano carbonaceous materials where fibrous and sheet-like morphologies are found to be of value. When considering this, it can be suggested that future studies should exploit the combined effect of different carbonaceous materials. An interesting and important aspect deduced from previous work is that the full exploitation of HAp-carbonaceous structures-based composites has still not been achieved yet. This is because modifications in both the HAp and carbonaceous components, like functionalisation and mineralisation, still need to be considered to a greater extent to further improve the properties of the resulting composites. Finally, it is also recommended to utilise other carbonaceous structures, like carbon nanobuds (its structure is a like a hybrid of fullerene and CNT and it appears that a fullerene-like bud is attached to the wall of CNT), carbon nanoform, Q-carbon (non-crystalline carbon, having mixed sp^2^ and sp^3^ bonding), carbyne (long chains of carbon), and lonsdaleite (hexagonal diamond). In this way, a new generation of biomaterials can be produced, which greatly improves on previous ones.
